# Supplementary material for: Cytoplasmic and Nuclear Forms of Thyroid Hormone Receptor β1 Are Inversely Associated with Survival in Primary Breast Cancer
Source: Int J Mol Sci. 2020 Jan 3;21(1):330. doi: 10.3390/ijms21010330 (PMC6981495; doi:10.3390/ijms21010330)
Supplement: Supplementary file 1 [file ijms-21-00330-s001.pdf]

Supplementary materials

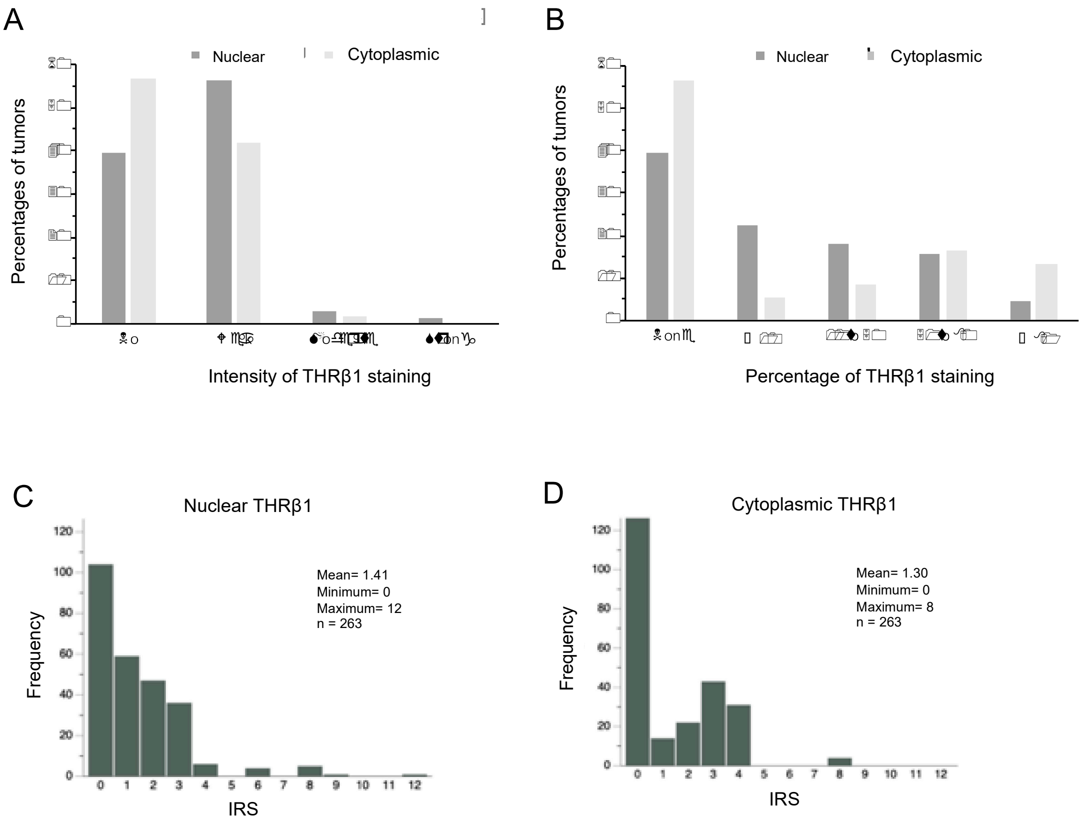

Figure S1. Distribution of intensity and percentages, and IRS frequency, of the nuclear and cytoplasmic THRβ1 stainings. The percentage of tumors with each intensity category (**A**) and percentage range (**B**) used for the IRS calculation are presented according to either the nuclear or cytoplasmic stainings of THRβ1. Distribution of IRS is represented according to either the nuclear (**C**) or cytoplasmic (**D**) stainings of THRβ1.

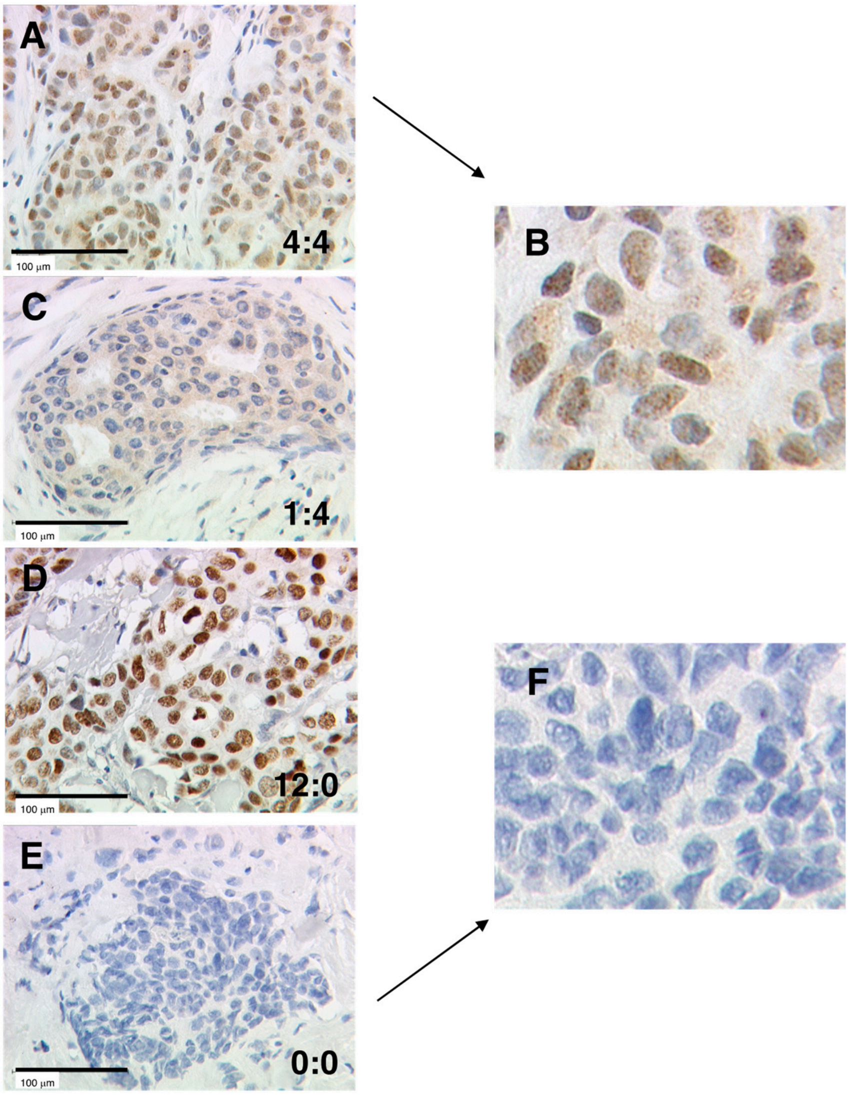

Figure S2. Immunohistochemical staining of THRα2 in BC samples. THRα2 staining is illustrated for 4 patients with examples of null or high expression. Samples A and E are enlarged in panels B and F respectively. Nucleo:cytoplasmic IRS (immunoreactive score) ratios are indicated in each photomicrograph (25× magnification) and scale bar equals 100 µm.

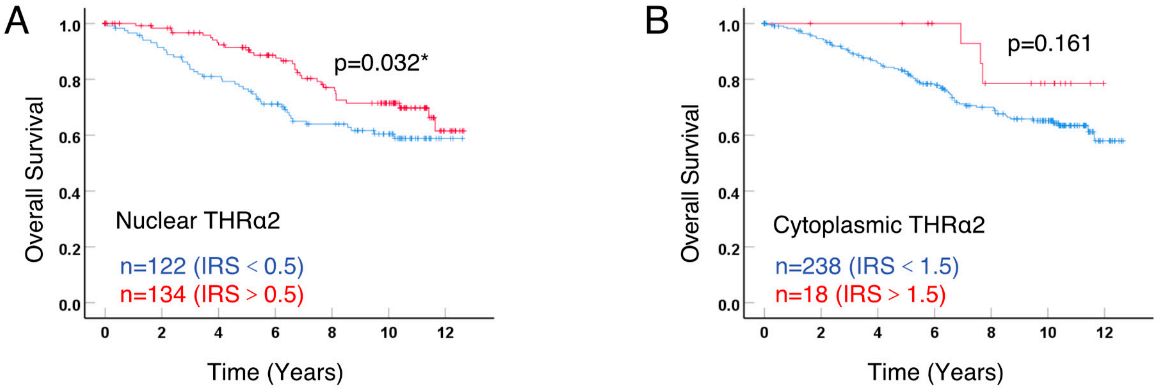

Figure S3. Kaplan-Meier analysis according to nuclear and cytoplasmic THRα2 expression. Overall survival (OS) curves are presented according to THRα2, with either the nuclear (**A**) or cytoplasmic (**B**) expression. The optimal IRS cut-off values with the number of cases for each group are indicated in each graph. Statistical significance is shown as *p*-value from log-rank test (*: $p < 0.05$).

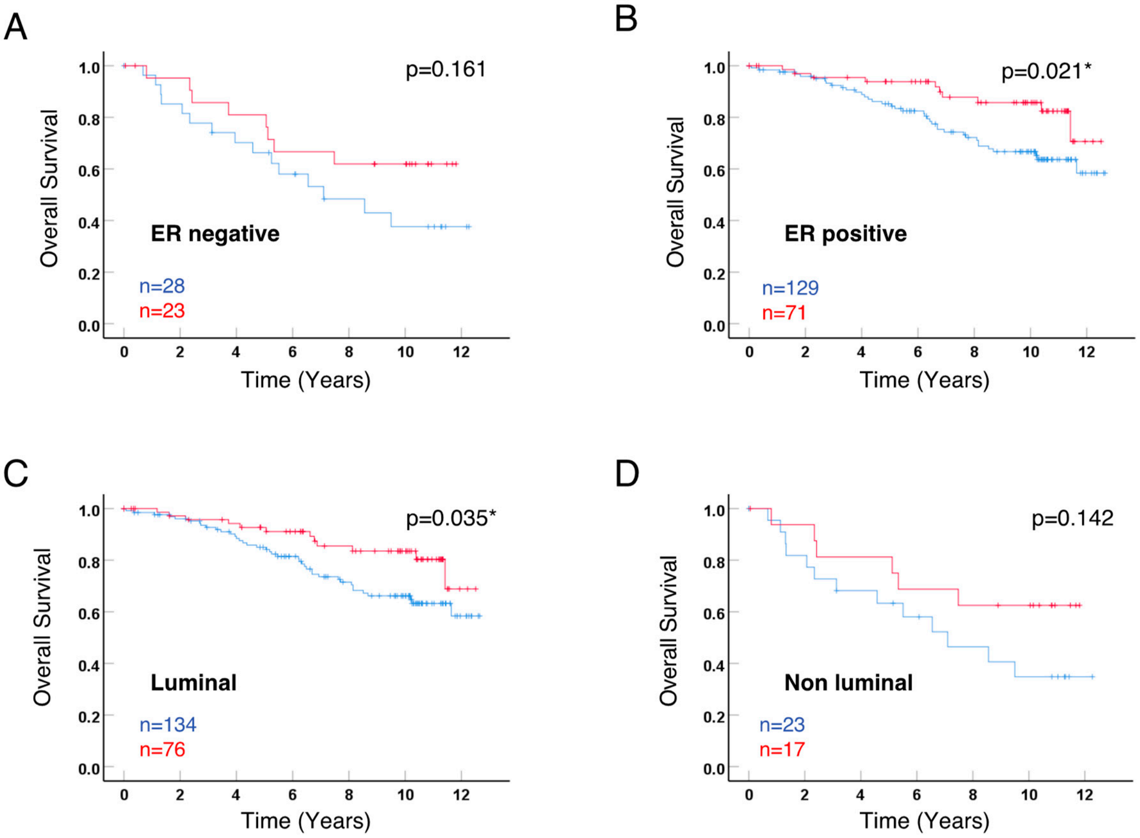

Figure S4. Kaplan-Meier analysis according to cytoplasmic THRβ1expression in ER positive vs negative and luminal vs non luminal sub-groups of patients. Overall survival (OS) curves are presented according to cytoplasmic THRβ1, for either ER negative (**A**) or positive (**B**) sub-groups of patients, and for either luminal (**C**) or non-luminal (**D**) sub-groups. The optimal IRS cut-off value of 1.5 was used and the number of cases for each group are indicated in each graph. Statistical significance is shown as p-value from log-rank test (*: $p < 0.05$).

Table S1. Distribution of the tumors with nuclear and/or cytoplasmic positive THRβ1 expression.

**Nuclear**	**Cytoplasmic**	n	%
Negative	Negative	86	32.7
Positive	Negative	63	23.9
Negative	Positive	18	6.8
Positive	Positive	96	36.5

Table S2. Distribution of THRα2 expression.

	Nuclear	Cytoplasmic
Mean IRS±SE	1.67±1.47	0.21*±0.04
Median IRS	1	0
IRS range	0-12	0-4
Negative expression**	120 (45.63%)	236 (89.73%)
Positive expression**	143 (54.37%)	27 (10.27%)

* Correlations were statistically significant for p<0.05 (*), using Spearman-Rho-Test using mean bilateral analysis; ** Negative and positive expression were respectively defined as IRS = 0 and IRS >0.
